# Isolated Third Cranial Nerve Palsy As the Initial Presentation of Primary Central Nervous System Lymphoma: A Rare Case

**DOI:** 10.7759/cureus.75715

**Published:** 2024-12-14

**Authors:** Nur Fadilah Azhani Mohammad, Noor Huda Abdul Wahab, Ng Kwang Sheng, Azma Azalina Ahmad Alwi, Rosiah Muda

**Affiliations:** 1 Department of Ophthalmology, Hospital Raja Perempuan Zainab II, Kota Bharu, MYS; 2 Department of Ophthalmology, Hospital Sultanah Nur Zahirah, Kuala Terengganu, MYS; 3 Department of Ophthalmology and Visual Science, School of Medical Sciences, Universiti Sains Malaysia, Kubang Kerian, MYS

**Keywords:** isolated third cranial nerve palsy, neurolymphomatosis, non-hodgkin lymphoma, pcnsl, pvrl

## Abstract

This is an unusual case of primary central nervous system lymphoma (PCNSL) with isolated third cranial nerve palsy as the initial manifestation. Neurolymphomatosis (NL) is a rare manifestation of PCNSL. While NL is a rare manifestation of PCNSL, primary vitreoretinal lymphoma (PVRL) can be the presenting feature or a later-involved manifestation. We present an unusual case of PCNSL with the isolated third cranial nerve palsy as the initial manifestation, which mimics the surgical third cranial nerve palsy. The patient developed PVRL subsequently and succumbed to the disease not long after the presentation.

## Introduction

There are two types of central nervous system lymphoma, namely primary and more commonly the secondary type [1]. Primary central nervous system lymphoma (PCNSL) is a rare, aggressive non-Hodgkin lymphoma that involves the brain, spine, cerebrospinal fluid, and eyes [2]. It accounts for about 3-4% of newly diagnosed intracranial neoplasms and affects males more than females [3,4]. The prevalence rises with increasing age, particularly, in patients of 65 years old and older [4,5]. PCNSL can affect both immunocompromised and immunocompetent hosts [6]. The neurological manifestations of patients with PCNSL are focal neurological deficits (70%), mental and behavioral changes (43%), symptoms of increased intracranial pressure (33%), and seizures (14%) [7]. Neurolymphomatosis (NL), on the other hand, is a condition characterized by nerve infiltration by neoplastic cells in hematological malignancies. It is a rare clinical manifestation of non-Hodgkin lymphoma that involves cranial nerves, peripheral nerves, and nerve roots or plexus [8]. We present a rare case of PCNSL with unilateral isolated third cranial nerve palsy as the initial presentation and subsequently developed intraocular involvement.

## Case presentation

**Figure 1:**
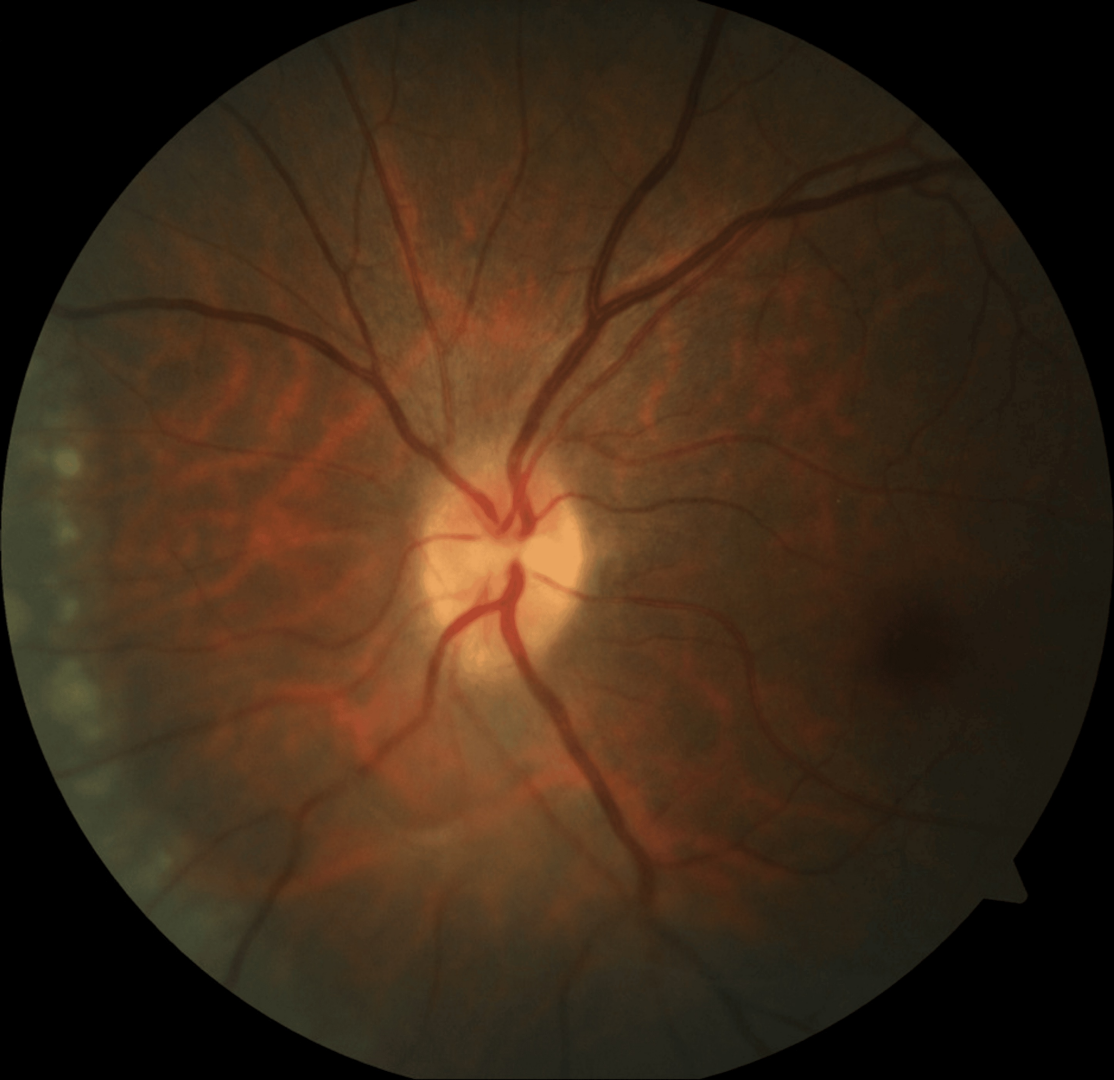
Fundus photo of the left eye shows optic disc swelling.

**Figure 2:**
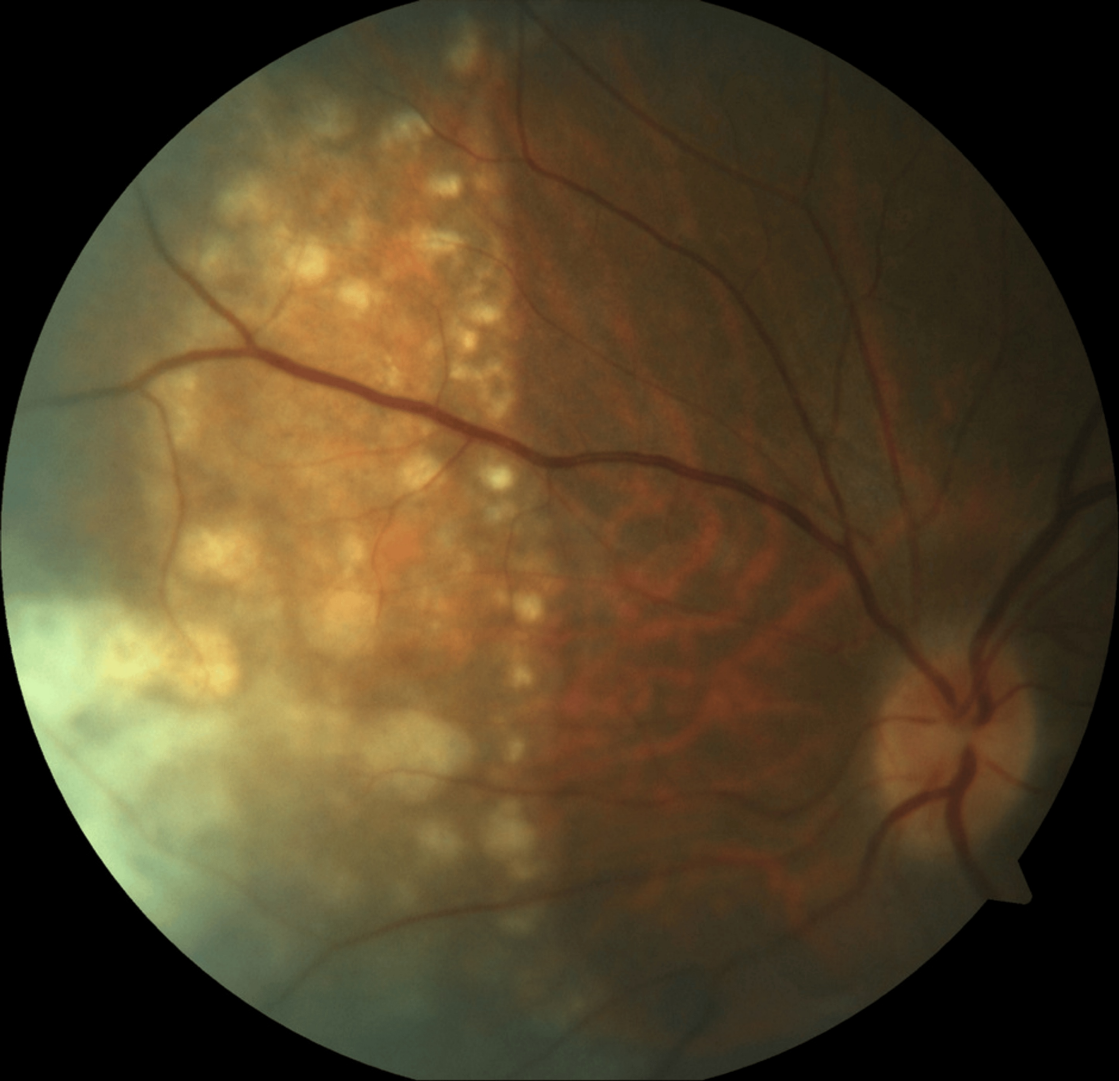
Fundus photo of the left eye shows multiple chorioretinal lesions nasal to the optic disc.

**Figure 3:**
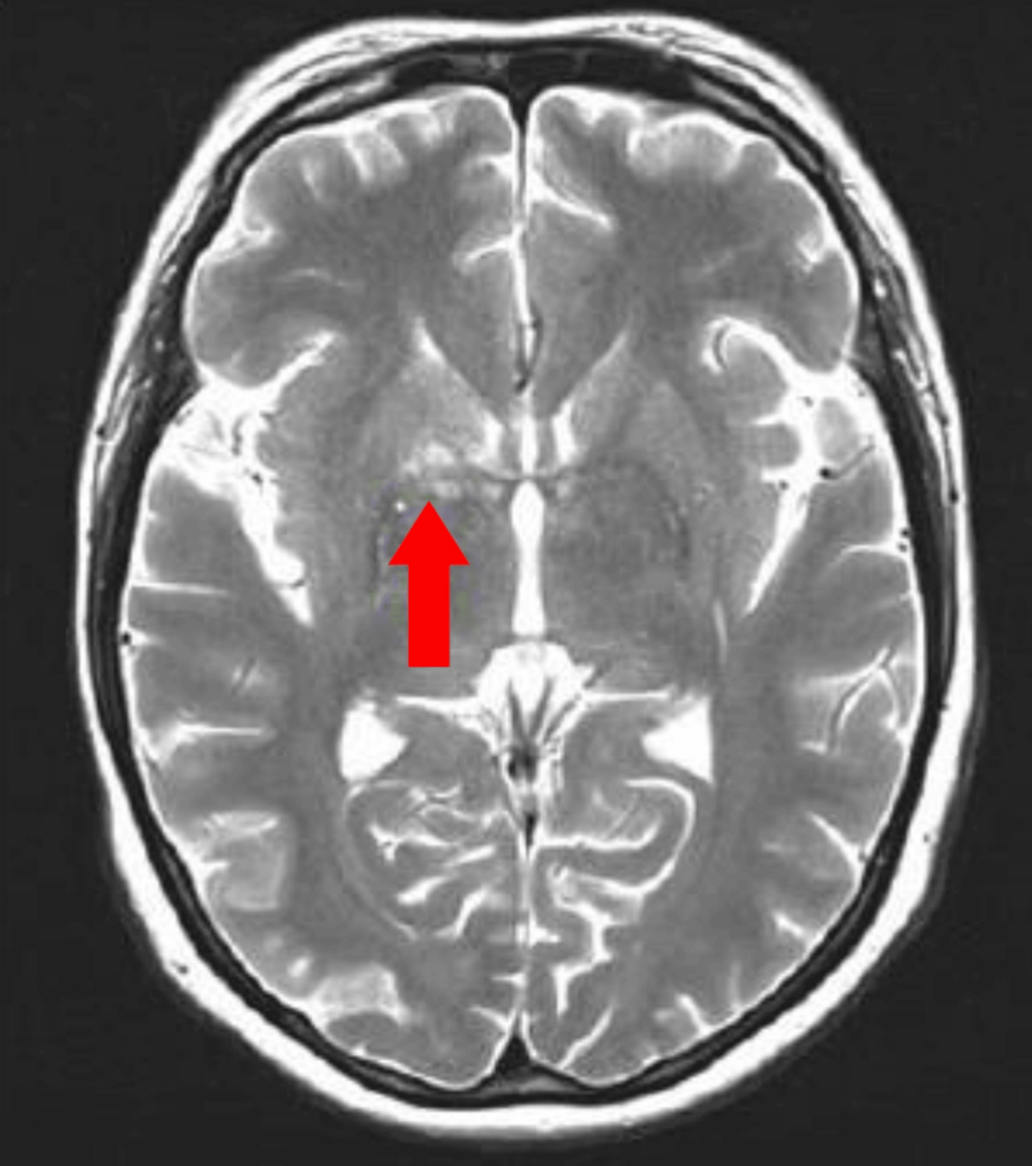
T2-weighted MRI of the brain shows hyperintense lesions at the right globus pallidus (red arrow).

**Figure 4:**
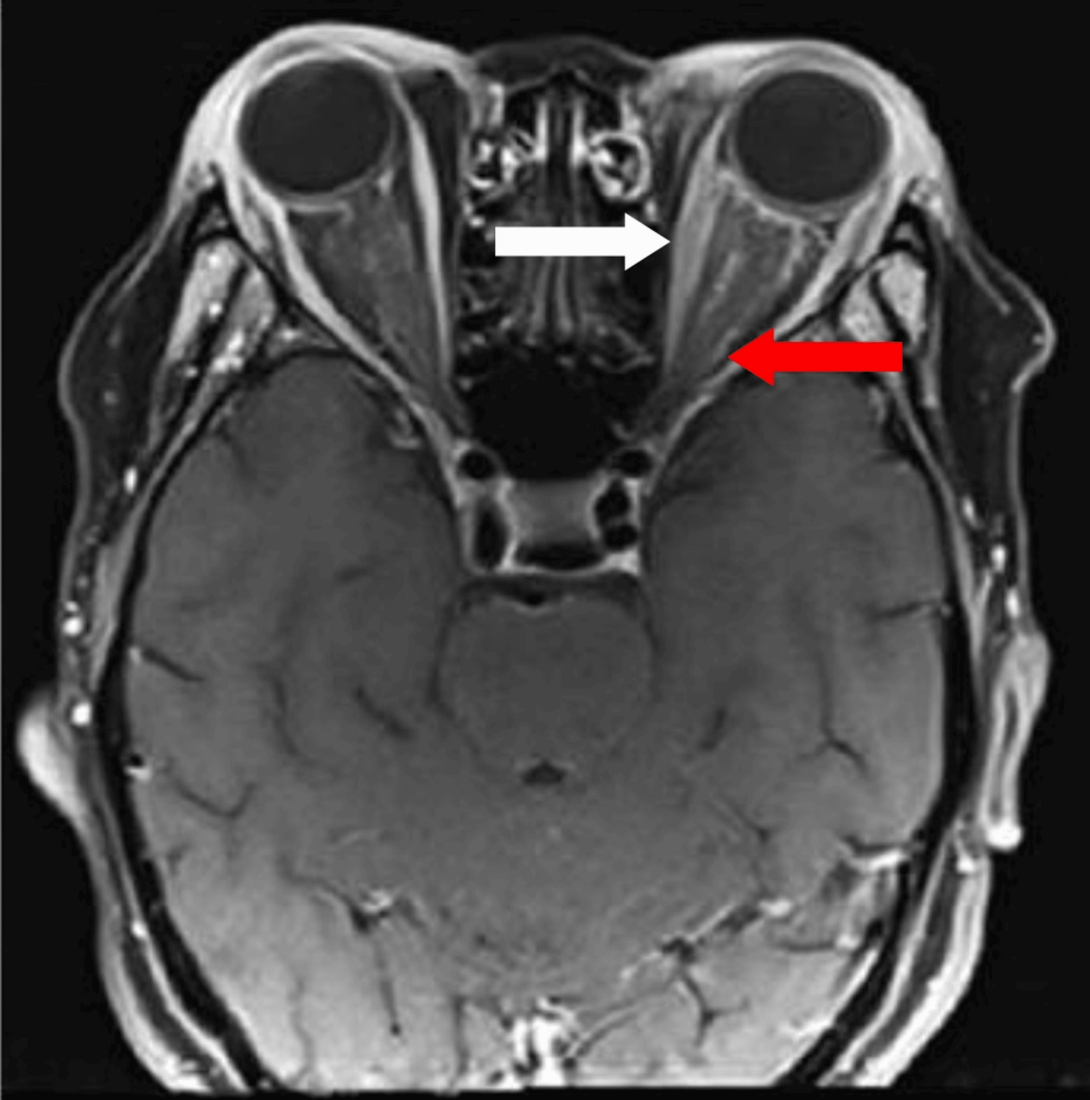
Gadolinium contrast MRI of the brain shows the bulky left optic nerve with perineural enhancement (red arrow) and bulky left medial rectus muscle (white arrow).

A 42-year-old woman with no known medical illness presented with sudden onset of right ptosis for two days associated with diplopia, headache, and vomiting. On examination, there was right complete ptosis, the right eye deviated outward with ophthalmoplegia other than abduction, the right eye pupil was dilated, the relative afferent pupillary defect (RAPD) was negative, and visual acuity for both eyes was 6/6. Other anterior segment assessments and fundus examinations were unremarkable. The initial diagnosis of right surgical third cranial nerve palsy was made. She underwent plain computed tomography (CT) of the brain but there was no obvious space occupying the lesion. The patient requested discharge at her own risk when further investigations, such as CT angiography to rule out a posterior communicating artery aneurysm, were planned. However, she presented again two months later with left upper limb weakness and numbness over bilateral upper and lower limbs. On examination, right complete ptosis persisted, but the extraocular movement had worsened to complete right ophthalmoplegia and limited left abduction. Visual acuity of both eyes had reduced slightly to 6/9. Fundus examination of the left eye showed vitritis 1+, optic disc swelling, and multiple chorioretinal lesions nasal to the optic disc (Figures 1, 2). MRI of the brain showed multiple T2-hyperintense lesions at the body and splenium of the corpus callosum, right superior cerebellar peduncle, and right globus pallidus (Figure 3). Left extraocular muscles were bulky and left optic nerve was bulky with perineural enhancement (Figure 4). Infective screening revealed positive HIV status with a CD4 count of 141 cells/mm^3^. Syphilis and tuberculosis screening were negative. She was referred to neurosurgery, and an image-guided biopsy was performed over her right globus pallidus lesion. Histopathological examination reported diffuse large B-cell lymphoma. However, the patient deteriorated and succumbed to death 10 days after the biopsy.

## Discussion

Although PCNSL can affect both immunocompetent and immunocompromised patients, congenital or acquired immunodeficiency such as AIDS or transplantation has been identified as the risk factor for the development of PCNSL [7]. Our patient had been diagnosed with retroviral disease (RVD), but she denied any high-risk behavior and informed us that her husband passed away 10 years ago due to brain cancer rather than RVD.

NL is rare and contributes to about 3% of newly diagnosed cases of non-Hodgkin lymphoma [9]. A study by Petluri et al. found that NL can present in four manifestations, namely painful polyneuropathy or polyradiculopathy (commonest), cranial neuropathy (20%), painless neuropathy, and peripheral mononeuropathy. The most common cranial nerves affected are the facial nerve, abducens nerve, oculomotor nerve, and trigeminal nerve in descending order [10]. Our patient presented with right complete oculomotor nerve palsy and subsequently her bilateral abducens nerves were also affected.

Primary intraocular lymphoma, known as primary vitreoretinal lymphoma (PVRL), is a rare subtype of PCNSL that affects the vitreous, retina, and optic nerve head. It can be the presenting manifestation or, more commonly, occur alongside brain lymphoma. Around 15-25% of patients with PCNSL are found to have PVRL at some point [11]. On the contrary, 56-90% of patients with PVRL have CNS involvement [12]. During the second presentation, our patient developed left eye PVRL with vitritis, chorioretinitis, and optic neuritis. MRI had also reported simultaneous brain involvement. It needs to be distinguished from systemic lymphoma with ocular involvement. Most patients with PVRL are over 50 years old with equal gender distribution. Our patient was much younger with the age of 42 years old at diagnosis. It could be probably due to her underlying immunodeficiency.

Although there is no pathognomonic imaging finding for PCNSL, MRI typically shows supratentorial lesions that are deep or located in the periventricular areas, with the most common locations being the cerebral hemispheres, basal ganglia, and corpus callosum [13,14]. Our patient showed similar involvement with lesions over her corpus callosum, right globus pallidus (part of basal ganglia), and right superior cerebellar peduncle.

PCNSL is a highly aggressive malignancy and over 90% of the cases are diffuse large B-cell lymphoma. Other histopathological types are Burkitt lymphoma, mantle B-cell lymphoma, and T-cell lymphomas [15]. It corresponds to our patient histopathological report.

The long-term prognosis for PCNSL is poor, with a five-year survival rate of approximately 30%, and the majority of patients would quickly succumb to death if their brains are involved and left untreated [15]. A study by Grisariu et al. concluded that the median survival from diagnosis of NL was about 10 months [8]. Our patient deteriorated and succumbed to the disease 10 days after the biopsy.

## Conclusions

Our case highlights that NL, a rare involvement in PCNSL, could be the presenting feature of PCNSL, which itself is also a rare disease. Additionally, it emphasizes the challenges in making the diagnosis, as the patient presented with the initial manifestation of right complete third cranial nerve palsy with pupil involvement. This condition could mimic the typical presentation of surgical third cranial nerve palsy, which may suggest intracranial lesions, such as a posterior communicating artery aneurysm. Furthermore, apart from anisocoria, other ocular and systemic examinations were unremarkable. On the other hand, the patient requested discharge at her own risk when further investigations were planned.
